# Influenza (H1N1) 2009 Outbreak and School Closure, Osaka Prefecture, Japan

**DOI:** 10.3201/eid1510.091029

**Published:** 2009-10

**Authors:** Ryosuke Kawaguchi, Masaya Miyazono, Tetsuro Noda, Yoshihiro Takayama, Yasunori Sasai, Hiroyasu Iso

**Affiliations:** Osaka Prefectural Government, Osaka, Japan (R. Kawaguchi, M. Miyazono, T. Noda, Y. Takayama, Y. Sasai); Osaka University Graduate School of Medicine and Public Health, Osaka (H. Iso)

**Keywords:** Influenza, H1N1, outbreaks, schools, mitigation, closure, Japan, viruses, letter

**To the Editor**: The Osaka Prefectural Government, the third largest local authority in Japan and comprising 43 cities (total population 8.8 million), was informed of a novel influenza outbreak on May 16, 2009. A high school submitted an urgent report that ≈100 students had influenza symptoms; an independent report indicated that a primary school child also showed similar symptoms.

The Infection Control Law in Japan requires that all novel influenza cases diagnosed by physicians and confirmed by laboratory test results be reported to public health centers. Influenza A pandemic (H1N1) 2009 infection was first detected in 2 students in the same high school on May 11, 2009, followed by an outbreak in a high school in city A in northern Osaka Prefecture (Appendix Table). Two days later on May 13 in city B in middle Osaka Prefecture, a primary school student and a junior high school student were found to be infected. Infections were also detected among school children in 6 other cities on May 14; six parents of students from the first outbreak school were also infected.

We obtained anecdotal information that influenza seemed to be transmitted from infected students in the first high school outbreak to students in other schools either because students had siblings who attended other schools or students were part of the same extracurricular clubs and cram schools (lessons after school to supplement schoolwork managed by a private company). Therefore, we concluded that the influenza (H1N1) 2009 virus was spreading widely to other schools and communities and that school closures would be necessary (*1*,*2*).

The governor of Osaka decided to close all 270 high schools and 526 junior high schools in Osaka Prefecture from Monday, May 18, to Sunday, May 24, following the weekend days of May 16 and 17 observed at most schools. Students were ordered to stay at home (*3*). Most nurseries, primary schools, colleges, and universities in the 9 cities with influenza cases voluntarily followed the governor’s decision. Antiviral drugs were prescribed by local physicians to almost all students with confirmed infection; families were given these drugs as a prophylactic measure. Family members of an infected student were also strongly encouraged to stay home at least 7 days after the student’s symptoms had disappeared. Most newspapers and radio and television stations began reporting the outbreak on May 16, and a national campaign emerged in which facemask use was recommended to the public along with good hygiene practices such as hand washing and gargling.

After the school closures, the number of newly reported cases declined rapidly from 30 cases on May 17 to none by May 25. During that time, 13 schools reported only 1 new case each. After May 25, although no new cases were found among students, some sporadic cases were identified among adults by the end of May. From June 1 through 22, twenty-five sporadic cases (of which 19 had become infected overseas) were reported, but no further outbreaks were reported in the schools.

Since June 23, smaller school outbreaks have occurred in cities in southern Osaka Prefecture. The government decided not to conduct the prefecture-wide school closure for these outbreaks. Instead, the decision regarding school closure was left to each school’s administrator. The prefecture-wide school closure strategy may have had an effect on not only the reduction of virus transmission and elimination of successive large outbreaks but also greater public awareness about the need for preventive measures.

## Supplementary Material

Appendix Table Number of confirmed cases of influenza A pandemic (H1N1) 2009 virus infection by school and onset date in Osaka Prefecture, Japan, May 11-31, 2009*
