# Supplementary material for: Influenza (H1N1) 2009 Outbreak and School Closure, Osaka Prefecture, Japan
Source: Emerg Infect Dis. 2009 Oct;15(10):1685. doi: 10.3201/eid1510.091029 (PMC2866419; doi:10.3201/eid1510.091029)
Supplement: Appendix Table — Number of confirmed cases of influenza A pandemic (H1N1) 2009 virus infection by school and onset date in Osaka Prefecture, Japan, May 11-31, 2009* [file 09-1029_appT-s1.pdf]

Appendix Table. Number of confirmed cases of influenza A pandemic (H1N1) 2009 virus infection by school and onset date in Osaka Prefecture, Japan, May 11–31, 2009*

		Date and day																				
		11	12	13	14	15	16	17	18	19	20	21	22	23	24	25	26	27	28	29	30	31	
City	Schools	M	T	W	T	F	S	S	M	T	W	T	F	S	S	M	T	W	T	F	S	S	Total
A	HS, JHS	2		6	10	19	18	16	13	5	1												90
	HS					1			1														2
	JHS						1	3	1		2												7
	PS												2										2
	HS							1															1
	JHS									1													1
	PS										1												1
	U										1												1
B	PS			1	1		2	3															7
	PS						1																1
C	HS				1																		1
	HS						1	1	2														4
D	JHS					1																	1
	HS							1	1														2
E	U					1																	1
	PS								1														1
	JHS										1												1
	JHS											1											1
	JHS													1									1
F	HS								1														1
G	JHS			1																			1
	HS					1		2		1													4
	JHS									1													1
	PS									1													1
	C													1									1
H	PS											1											1
I	U												1										1
Adults					2		1	3	2	3	3	2			2		1						19
Total		2	0	8	14	23	24	30	22	12	9	4	3	2	2	0	1	0	0	0	0	0	156

*PS, primary school; JHS, junior high school, H, high school; C, college; U, university. All case-patients were students except those in the Adults row. Gray shading indicates days of school closure.
